# Supplementary figures and images for: Proteomic analysis of hippocampus reveals metabolic reprogramming in a piglet model of mild hypoxic ischemic encephalopathy
Source: PLoS One. 2025 Apr 24;20(4):e0320869. doi: 10.1371/journal.pone.0320869 (PMC12021231; doi:10.1371/journal.pone.0320869)

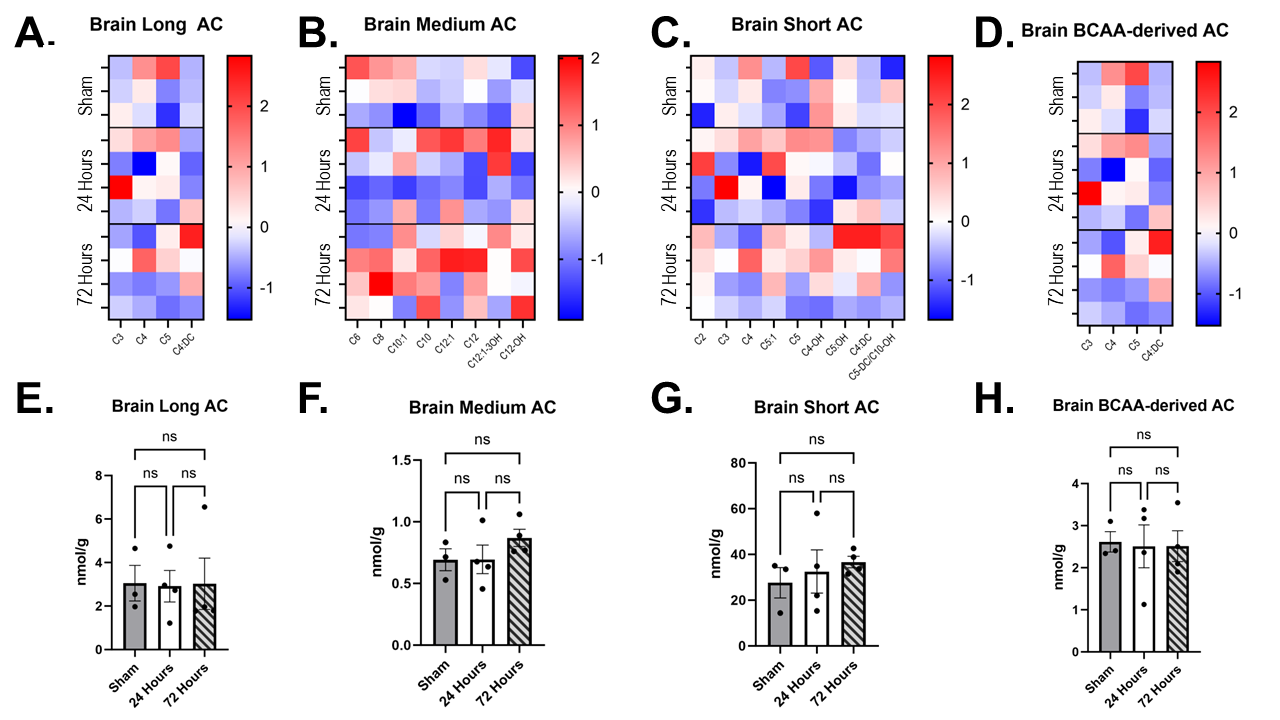

Supplement: S1 Fig — (A-D) Heat maps of z-scores for brain acylcarnitines (AC) are shown. Individual acylcarnitine species are denoted on the x-axis. (E-H) Mean +/− SEM for each acylcarnitine group. (TIF) [file pone.0320869.s002.tif]
